# How do care providers evaluate collaboration? - qualitative process evaluation of a cluster-randomized controlled trial of collaborative and stepped care for patients with mental disorders

**DOI:** 10.1186/s12888-021-03274-3

**Published:** 2021-06-08

**Authors:** Kerstin Maehder, Silke Werner, Angelika Weigel, Bernd Löwe, Daniela Heddaeus, Martin Härter, Olaf von dem Knesebeck

**Affiliations:** 1 Department of Psychosomatic Medicine and Psychotherapy, University Medical Center Hamburg-Eppendorf, Hamburg, Germany; 2 Institute of Medical Sociology, University Medical Center Hamburg-Eppendorf, Hamburg, Germany; 3 Department of Medical Psychology, University Medical Center Hamburg-Eppendorf, Hamburg, Germany

**Keywords:** Collaborative care, Stepped care, Mental health, Randomized-controlled trial, Implementation, Qualitative study, Process evaluation

## Abstract

**Background:**

Collaborative and stepped care (CSC) models are recommended for mental disorders. Their successful implementation depends on effective collaboration between involved care providers from primary and specialist care. To gain insights into the collaboration experiences of care providers in CSC against the backdrop of usual mental health care, a qualitative process evaluation was realized as part of a cluster-randomized controlled trial (COMET) of a collaborative and stepped care model in Hamburg (Germany).

**Methods:**

Semi-structured interviews were conducted with *N* = 24 care providers from primary and specialist care (outpatient psychotherapists and psychiatrists, inpatient/ day clinic mental health providers) within and outside of COMET at the trial’s beginning and 12 months later. Interviews were analyzed applying a qualitative structuring content analysis approach, combining deductive and inductive category development.

**Results:**

Usual mental health care was considered deficient in resources, with collaboration being scarce and mainly taking place in small informal networks. Within the COMET trial, quicker referral paths were welcomed, as were quarterly COMET network meetings which provided room for exchange and fostered mutual understanding. Yet, also in COMET, collaboration remained difficult due to communication problems, the unfavorable regional distribution of the COMET care providers and interprofessional discrepancies regarding each profession’s role, competencies and mutual esteem. Ideas for improvement included more localized networks, the inclusion of further professions and the overall amelioration of mental health care regarding resources and remuneration, especially for collaborative activities.

**Conclusions:**

The process evaluation of the COMET trial revealed the benefits of creating room for interprofessional encounter to foster collaborative care. Despite the benefits of faster patient referrals, the COMET network did not fulfill all care providers’ prior expectations. A focus should be set on interprofessional competencies, mutual perception and role clarification, as these have been revealed as significant barriers to collaboration within CSC models such as COMET.

**Trial registration:**

The COMET trial (Collaborative and Stepped Care in Mental Health by Overcoming Treatment Sector Barriers) has been registered on July 24, 2017 under the trial registration number NCT03226743.

**Supplementary Information:**

The online version contains supplementary material available at 10.1186/s12888-021-03274-3.

## Background

Mental disorders are high in prevalence [1], pose a significant burden for those affected [2] and entail high costs both in health care and economically [3, 4]. Comorbidity rates between mental disorders are substantial, with an estimated 44% of patients having two and 22% having three or more mental conditions [5]. Most patients with mental disorders are first and partly exclusively cared for in primary care settings [6]. Guidelines in the UK, the Netherlands and Germany recommend collaborative and stepped care as models of health care provision for mental disorders [7–9]. While collaborative care models differ in their particular designs, the essential elements are: 1) team-driven care, i.e. care is provided in a coordinated way by a multidisciplinary group of health care providers, 2) population-focused, i.e. care aiming at a defined group of patients, in this case those with mental disorders, 3) measurement-guided, i.e. care being guided by systematic patient-oriented outcomes and 4) evidence-based care [10]. Stepped care further adds evidence-based care pathways with accurate alignment of care intensity based on illness severity, thus offering the lowest step of care intensity required. Moreover, the adaptation of care intensity is assured by systematic monitoring and subsequent stepping up or down or maintaining of care intensity [11]. 

Collaborative care models have been proven effective for a range of mental disorders [12–14].

To assess the effectiveness of a collaborative and stepped care (CSC) model for patients with the most common mental disorders (depression, anxiety, somatoform and alcohol-related disorders [15]), we conducted a prospective cluster-randomized controlled trial (RCT) in primary care. Details on the *Collaborative and Stepped Care in Mental Health by Overcoming Treatment Sector Barriers* (COMET, NCT03226743) can be found in the study protocol [16]. A collaborative care network of primary care physicians (PCPs) and mental health professionals (= MHPs: registered outpatient psychotherapists and psychiatrists, inpatient and day-clinic mental health providers) was built in the metropolitan area of Hamburg, Germany. To facilitate collaborative care, we established an online scheduling platform, where PCPs could directly schedule psychotherapist or psychiatrist appointments with MHPs from the network for their patients, thus lowering the threshold for psychotherapeutic and psychiatric care. Furthermore, we encouraged and financially reimbursed exchange between PCPs and MHPs, both regarding shared patients (e.g. via phone calls or specific treatment reports) and by offering quarterly CME-accredited network meetings that combined professional training and interprofessional dialogue. Furthermore, recommendations of stepped care pathways were given based on the clinical diagnosis of the PCPs and following an evidence-based algorithm. The COMET CSC model distinguishes itself from previous CSC models by implementing the aforementioned innovations in the existing structures of usual care with its small-practice structure in Germany, without adding new care professional roles or external facilitators, e.g. study nurses or care managers. Moreover, it explicitly accounts for the high rate of comorbidity between mental disorders by addressing the four most common mental disorders, which frequently co-occur and pose challenges to differential diagnostics especially in primary care.

The effectiveness and implementability of complex care models like the CSC model in COMET depend on the ability and willingness of the involved care providers to adopt new tasks and to collaborate. Thus, it is indispensable to consider their perspectives. Previous studies on CSC models in primary care have revealed different challenges for collaboration. Facilitators for collaboration were identified in clear care provider roles, sufficient coordination and personal contacts [17–20]. In contrast, care providers in CSC studies criticized when collaboration came along with too many and/or not sufficiently remunerated tasks, against the background of already high workload and little treatment capacities [19–21]. Difficulties arose when interests of care providers diverged too largely [17, 19] and when communication, interaction and/or feedback pathways were deficient, both structurally and on content-level [20–22].

Considering these prior study results, we conducted a process evaluation alongside the COMET-trial. This evaluation focused on collaboration experiences between PCPs and MHPs, both within and outside the COMET network. In addition to the existing research, we explicitly accounted for the usual mental health care situation at baseline by inquiring on the collaboration experiences of the care providers in usual care before trial implementation. Also, we included a broader range of common and often comorbid mental disorders than in most disorder-specific trials, in order to mirror prevalence within the general population and to build a closer link between trial conditions and usual mental health care. Moreover, as PCPs and MHPs in Germany predominantly work in small practices, we wanted to shed light on the collaboration challenges in outpatient care without co-location, which may differ from CSC models implemented in hospitals or health organizations [23]. Furthermore, we focused on potential difficulties in interprofessional relations, as these have been considered a barrier to collaboration beforehand. The research question was: How do PCPs and MHPs experience and evaluate collaborative care within the COMET study against the background of usual mental health care?

## Methods

### Design

To thoroughly evaluate the COMET trial, a complex and theory-based process evaluation was developed [24–26], including short questionnaires on demographic and professional characteristics and interviews with health care providers at two time points. The implementation framework that served as a basis for the development of the interview guides was Greenhalgh’s model of Diffusion of Innovations in Service Organizations [24], complemented by the Medical Research Council guidance on process evaluations of complex interventions [25] and May’s Normalization Process Theory [26]. These frameworks were chosen to assure a thorough evaluation of a complex care model, whose implementation depends on a wide range of influencing variables, such as the specifics of the COMET CSC model, the participating care providers or the general health care system. The main research foci for the overall process evaluation were 1) aspects of the innovation, i.e. the COMET CSC model, 2) adoption and assimilation processes regarding the uptake of the CSC model by both care providers and patients, 3) communication and networking, 4) system context, e.g. the general health care system and 5) the study implementation process, e.g. regarding study information or incentives. In this paper, we focus on the care providers’ experiences with and evaluation of collaboration, as this is a key feature of CSC models. While this touches on every of the aforementioned categories, the focus mainly related to the category of communication and networking, entailing questions such as “How did communication within the COMET network work?” (see Additional file 1). The qualitative approach was chosen to allow for an in-depth understanding of the experiences and evaluations of both PCPs and MHPs. Reporting followed the COREQ checklist (Consolidated criteria for Reporting Qualitative research, [27]).

### Sampling and participants

All PCP interviewees took part in the COMET-trial, either having been randomized to the CSC or the treatment as usual (=TAU) group. As the MHPs in COMET were all members of the CSC group, we further approached mental health specialists outside the COMET network to gain insights into collaborative experiences outside the trial. One year later, only the PCPs and the MHPs within the COMET trial were interviewed again, as those outside the trial were not further influenced by the CSC model.

To reach an interview sample with heterogenic characteristics of the overall COMET care provider group, a purposeful sampling approach was adopted [28]. Sampling criteria for both PCPs and MHPs were the following, each in accordance with their respective share within the COMET-network: gender, socioeconomic status of the area of practice (as defined by the social monitoring report 2018 of the city of Hamburg [29]) and years of professional experience. As to the MHPs, interviewees were chosen from the three groups 1) outpatient psychotherapists, 2) outpatient psychiatrists and 3) inpatient/ day clinic mental health providers. Furthermore, main sampling criteria for MHPs were: professional background (medicine or psychology; both groups can qualify for outpatient psychotherapy in Germany) and the psychotherapeutic approach (either in psychodynamic or cognitive-behavioral psychotherapy; both approaches are reimbursable by health insurances in Germany [30]). Interviewees were approached by phone, mail or in person and gave informed consent. Sampling was stopped, when all sampling criteria were sufficiently represented and further interviews did not bring about significantly new insights. Overall, only two psychotherapists declined to participate due to lack of time. The final sample characteristics are depicted in Table 1.

**Table 1:** Characteristics of the COMET process evaluation interviewees

	Primary Care Physicians (PCPs)	Mental Health Professionals (MHPs)
COMET CSC group (n)	6	8
COMET TAU group (n)	6	–
Usual care outside COMET	–	4
Age (mean (min–max))	50.1 (31–64)	51.2 (34–70)
Sex (n)		
Female	7	8
Male	5	4
Working experience in years (mean (min–max))	16.9 (1–35)	*n* = 11 16.6 (2–34)
Socioeconomic status of practice location (n)		
Middle to high	9	11
Very low to low	3	1
Psychotherapeutic approach, if applicable (n)		n = 11
Cognitive-behavioral psychotherapy	–	6
Psychodynamic psychotherapy	–	5

*COMET* Collaborative and Stepped Care in Mental Health by Overcoming Treatment Sector Barriers (trial name)

*CSC* Collaborative and Stepped Care (intervention group within COMET)

*TAU* Collaborative and Stepped Care Treatment as usual (control group within COMET)

### Data collection

Semi-structured interviews were conducted at two time points during the COMET-trial: T1 at the beginning of the trial, T2 approximately one year after T1 to capture the changes within the CSC model, each incentivized monetarily. Based on research literature on implementation and previous CSC models [21, 24, 31] parallel interview guides were developed for both professional groups and time points of interviews (see Additional file 1). All interviews were conducted one-on-one between July 2018 and June 2020, mostly face-to-face in the care providers’ practices, during the coronavirus-pandemic via phone. The interviewing researcher (SW) was a female sociologist with prior experience and training in qualitative research, who had as well contact to the participants within the COMET study implementation (recruitment, study instruction and network meetings).

Interviews were audiotaped and transcribed verbatim while assuring pseudonymization regarding all identifying details, e.g. names or area of practice. Mean duration of interviews was 42 min.

### Analysis

The transcripts were analyzed by using the qualitative structuring content analysis [32–34], an approach aiming at extracting the core content of the data in a systematic, rule- and theory-based way. Analysis was realized by two female researchers with the following backgrounds: SW (see above) and KM, a PhD student and psychologist with prior experience and training in qualitative research who had as well contact to the participants within the COMET study implementation (recruitment, study instruction and network support and meetings).

The analytic process was managed using MAXQDA 18 (Verbi GmbH). Deductive categories were inferred from the interview guide, supplemented by inductive categories developed bottom-up from the data. Starting with deductive categories assured to map onto the theoretically derived evaluation foci, thus keeping track of a wide range of potential influences on overall study implementation and experiences. In this paper, we focus on the collaborative care evaluation within COMET, against the background of the evaluation of usual mental health care outside the trial. After coding the transcripts, the two perspectives of PCPs and MHPs were discussed and compared in recursive manner, as were the categories, their definitions and delineations until consensus was reached.

To further increase intersubjective comprehensibility and credibility [35], the process evaluation was discussed in a meeting of an interdisciplinary work group for qualitative methods and in an interdisciplinary meeting for psychosomatic research. Parts of the interviews conducted at T1 were presented orally to the interviewees at T2 both to approve our understanding of the interviewees’ statements and to build on these for possible changes at T2.

## Results

### Overview

**Fig. 1:**
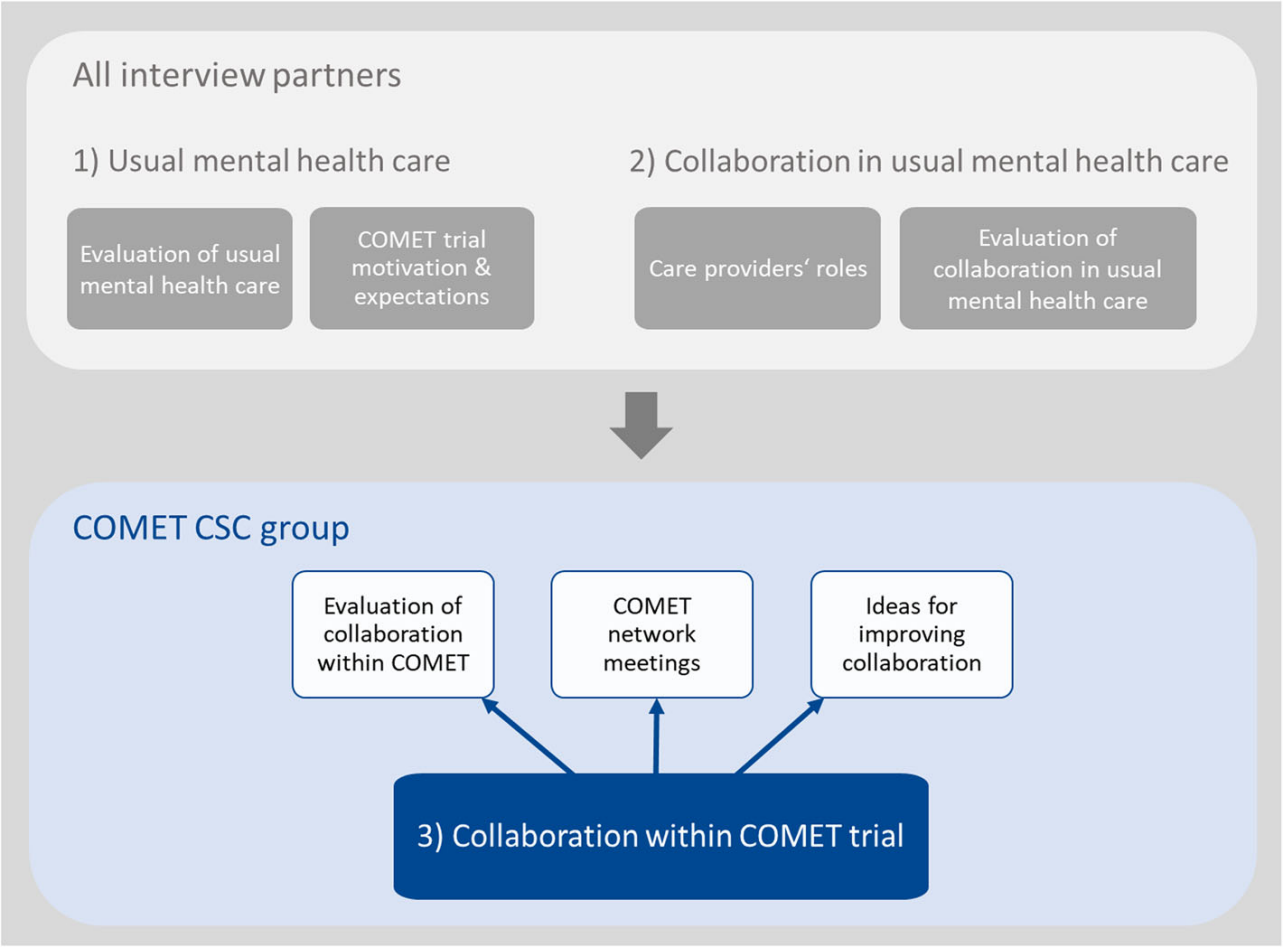
Main categories of the process evaluation on collaboration in the COMET trial. *COMET* Collaborative and Stepped Care in Mental Health by Overcoming Treatment Sector Barriers (trial name), *CSC* Collaborative and Stepped Care (intervention group within COMET)

Results on how PCPs and MHPs experience and evaluate collaborative care within the COMET study will be presented according to the identified main categories (Fig. 1). A summary of results with exemplary quotes can be found in Additional file 2. To set the baseline, the evaluation of the usual mental health care situation as judged by the interviewees is presented first, followed by their expectations with regard to the COMET trial (1). Second, the interviewees’ description of care providers’ roles and their evaluation of collaboration experiences in usual mental health care are depicted, as these influenced both trial motivation and collaboration evaluation within the COMET trial (2). Third, results with regard to collaboration within the COMET trial, the COMET network meetings, and ideas for improvement are elaborated on (3). Quotes were chosen as anchor citations for the categories and were edited for legibility and explanations have been added in square brackets where necessary. Quotes are identified by professional group (primary care physician (PCP) vs. mental health professional (MHP)), gender (female (f) vs. male (m)), T1 or T2 interview and number of participant.

#### Usual mental health care

The evaluation of usual mental health care at baseline and trial motivation and expectations as judged by the interviewees were a central part of the interviews conducted before starting the COMET trial (T1).

#### Evaluation of usual mental health care

Both PCPs and MHPs judged the mental health care offer in Hamburg as insufficient, even “dramatic” (MHP/f/T1/17), lacking in both outpatient and inpatient psychotherapeutic and psychiatric care, despite being “officially an area of overcare” (MHP/m/T1/24). While some interview partners recognized the even scarcer care offer in other areas in Germany and in comparable countries, distinctions were as well made regarding different districts of Hamburg and depending on type of mental illness and severity. PCPs herein differentiated their assessment, with care being especially wanting for patients with moderate to high illness severity outside of acute crises and for patients with substantial psychosocial problems:

“There’s a big gap for patients, where you think ‘He really suffers’ but if I admitted him to a psychiatric hospital, that would be too tough, he would benefit from outpatient care, but there’s no one [I could refer to].” (PCP/f/T1/9)

Besides the resulting mutual frustration and burden for patients and care providers, both PCPs and MHPs criticized the higher risk for illness chronification, comorbidities and long work-related disability as a result of long waiting periods for mental health care. Apart from the lack of time and resources in health care in general, one major deficit in mental health care was seen in a lack of interdisciplinary collaboration and communication, except for collaboration within local informal networks built over time (see section 2 below). A further barrier for mental health care and collaboration was seen in the ongoing stigmatization of mental disorders, with some MHPs and PCPs detecting a change towards more openness and acceptance within society. Yet, some PCPs questioned whether psychotherapy was necessary for all those asking for it, worrying that patients with mild disorders who “somehow find it quite nice to get to know themselves a little better” (PCP/m/T1/6) block the treatment resources needed for patients with higher severity. Judgements on the usual mental health care situation did not change or only to slight degrees when inquired again 1 year later (T2).

#### COMET trial motivation and expectations

Based on this overall evaluation on mental health care, the main motivation to participate in the CSC trial for both PCPs and MHPs was intensified collaboration in order to improve care for patients with mental disorders. This wish for fostered collaboration was equally expressed by participants in the TAU group. Especially for PCPs, it was of central importance to refer patients to psychotherapists and psychiatrists quicker than in usual care and to have more structured care pathways. Furthermore, both provider groups were interested in interdisciplinary exchange and further training in mental health care. While financial incentives and credits for continuing medical education within COMET did not play a major role for motivation in either group, they were still welcomed. To a lesser extent, interest in research and previous trial experiences were mentioned as reasons to take part in COMET.

### Collaboration in usual mental health care

After presenting the care providers’ roles, as depicted by the interviewees, the focus is set on collaboration experiences before and outside the COMET trial, mainly captured in T1 interviews.

#### Care providers’ roles

PCPs’ view on their role in health care was one of being a “pilot” (PCP/m/T1/4) in health care, based on being the initial point of contact, their trustful relationship with most of their patients and their coordinative function. They cited a multitude of tasks they considered themselves responsible for, including differential diagnostics, low-level and low-threshold interventions (especially supportive regular consultations to bridge the gap until further treatment and during crises), medication, referrals, motivation for psychotherapy, relapse prevention, sick certification, etc. When caring for patients with mental disorders, most referred to their lack of time and the high number of patients as major barriers. Especially those PCPs, who acknowledged their relative lack of competence in mental health care, were keen on referring patients to MHPs. Others alluded to their years of professional experience and to further training they underwent in mental health care, considering themselves qualified for treating mild to moderate mental disorders:

“I don’t hesitate working with psychotropic drugs, to ease the access for patients. That’s not too complicated. All that diagnostics of depression, all these diagnostic criteria, be it mild, be it moderate, […] that’s what we usually start with here and where we offer at least some kind of medication.” (PCP/m/T1/8).

Concerning their own role in mental health care, MHPs emphasized their competence for differential diagnostics, psychoeducation and treatment (psychiatric, psychotherapeutic or psychosomatic) of mental disorders. Yet, patients suffering from addiction were often declined or only accepted as clients if they had undergone previous addiction treatment. MHPs with a focus on psychotherapy underlined the importance of mutual fit and patient motivation, in contrast to settings such as primary care. Thus, they justified being selective with patient choice: “We work with our relationship, it is impossible that I get forced to take just any patient” (MHP/f/T1/17). MHPs considered themselves privileged due to their extended session time and criticized that PCPs lacked both time and financial remuneration for communication with patients. All the more as PCPs were deemed to be an important first and partly the only point of contact for patients with mental disorders, crucial for identifying mental health problems and paving the way for psychotherapy. However, PCPs’ interest and knowledge in mental health were considered quite diverse and overall limited. Yet, extensive knowledge in mental health was not expected from PCPs, as MHPs saw this as their area of competence and disapproved of too many tasks being shifted to PCPs.

Recurring mutual prejudices between the different professional groups emerged, combined with “partly little (…) empathy or sympathy or willingness to imagine what the other one experiences” (MHP/m/T2/14). One of these professional gaps showed up between psychotherapists with psychological background and physicians of non-psychotherapeutic specialties: “I still have the feeling that there is not much appreciation. And that most physicians don’t even know what we do and how much it matters to patients that there is someone listening” (MHP/f/T1/19). These experiences were partly reflected on the other side, as one PCP described a fairly good exchange with psychiatrists while with psychotherapists it felt like there is “some kind of curtain between [them and us]” (PCP/f/T1/9). Yet, only one participant generally challenged the mental health care system with its sector and specialty boundaries: „There is quite an arrogance in Germany among the specialists and among the outpatient psychotherapists as well” (MHP/f/T2/18).

#### Evaluation of collaboration in usual mental health care

Overall, collaboration in mental health care was deemed insufficient, mainly due to a lack of time, resources and financial remuneration. This entailed the risk of „psychotherapist and PCP not talking to each other […] causing wrong decisions: an antidepressant is not being prescribed or whatever” (MHP/m/T1/24). However, most PCPs and MHPs reported having at least some collaboration partners in their surroundings, ranging from sporadic contacts to mostly stable informal local networks built over time. Facilitators for such networks were, first and foremost, spatial proximity or sometimes shared practice, leading to shared patients. Besides the geographical adjacency, networks were further enabled by personal contacts arisen from either professional or private contexts (e.g. having worked at a clinic together), accumulated years of professional experience, being part of other professional networks (e.g. medical networks), political engagement on behalf of one’s profession and sometimes even coincidences. Close collaboration required interest in networking and proactive commitment, as one MHP explained: “I think it’s an illusion to create a network without this taking my time (…)” (MHP/f/T1/18).

When collaborating, both PCPs and MHPs welcomed the chance of short referral pathways and of coordinating and aligning care. Collaboration was even considered essential: „I think that the mental health care situation currently largely depends on whether you have a good professional network” (PCP/f/T1/10). The MHPs experienced a heightened trust in the adequacy of referral and the reliability of referred patients, when issued by a collaborating PCP. Learning from each other was considered a further benefit.

While collaboration within the established local networks was appraised, the collaboration experiences outside these networks revealed more difficulties. Against the background of overall limited time, resources and refund for collaboration, a core conflict was to reach each other, thus to establish personal contacts. The PCPs additionally disapproved the long waiting times for patients for mental health care and the lack of feedback from mental health care providers after having referred patients: “And then patients go [to a psychotherapist] and you never hear from them again. That’s the way it normally works” (PCP/f/T1/2). This points to another aspect that MHPs recognized: the differences between their work and a PCP setting, e.g. referral direction, information interest and the number of patients, linked to the number of potential collaborators. Collaboration for mental health care was further influenced by the intimacy of issues that arise especially in psychotherapy. Sharing this information with further care providers, such as the PCP, was partly disapproved of or only considered with “reservation and skepticism” (MHP/f/T1/15) on both the patients’ and the MHPs’ side. In addition, active collaboration was considered a tightrope walk between the personal responsibility of patients for their mental health treatment, their current ability for this and the extent to which the care providers should take over responsibility.

#### Collaboration within the COMET trial

Collaboration experiences within the COMET trial were discussed at T2 with the PCPs of the CSC group and the COMET MHPs. First, the participants’ evaluation of collaboration within the COMET trial will be presented, followed by the evaluation of the quarterly network meetings and, finally, ideas for improvement will be summarized. Due to the trial structure, collaboration mainly took place between PCPs and psychotherapists, to a lesser extent with psychiatrists and rarely with the participating inpatient facilities.

#### Evaluation of the collaboration within COMET

Collaboration within COMET was welcomed as a chance for increased personal contacts, mutual understanding and estimation:

“I didn’t think about what a day looks like for a PCP, I was just irritated when I again tried to reach one for hours. (…) Within the group work [at the network meetings] I understood some of the difficulties that PCPs have to struggle with in their everyday practice” (MHP/m/T2/14).

Changes were even transferred outside COMET, as one MHP claimed “It probably is more due to my approach, that I’ve become more open” (MHP/m/T2/20).

As for the patients, faster referrals to psychotherapy and psychiatric diagnostic and treatment sessions were enabled and considered beneficial, especially on the PCPs’ part. Psychotherapists themselves welcomed the chance for psychiatric appointments on short call. The online platform that allowed care providers to book appointments offered by the trial’s MHPs was valued by both professional groups and allowed the PCPs to pass short notes on a patient, although at times PCPs criticized a lack of appointments on offer. PCPs appreciated receiving more feedback than in usual care and being able to contact MHPs for professional inquiries. MHPs were generally satisfied with the patients that have been referred to them by the trial’s PCPs and partly perceived them even as more reliable. Yet, some MHPs experienced COMET-patients as less motivated and assumed this to be a downside of too quick referrals to psychotherapy. The COMET-patients’ reduced reliability was attributed to them having “clearly less appreciation for [a psychotherapy offer], probably because they got it so easily”. In contrast, patients in usual care, who usually have to endure long waiting times and need to invest effort for getting psychotherapy seem to “generally have already reflected [on their problems] a lot” (MHP/f/T2/17).

Despite these positive effects, the evaluation of the general collaboration intensity and sustainability was divergent among the professional groups. PCPs were mostly satisfied except for still perceived difficulties in communication and only partly improved feedback, while for part of the MHPs, the network did not match the interviewees’ prior expectations and wishes: “To be honest, this might change a little section but it doesn’t change anything in the general way of mental health care” (MHP/m/T2/13). Besides the persisting deficiencies in time and resources, the key reason for deficient collaboration was seen in the unfavorable distribution of network partners in the city of Hamburg: PCPs were located in various and partly peripheral districts whereas most psychotherapists were clustered in central quarters. The resulting long journeys for the trial patients to access psychotherapy impeded, in the MHPs’ eyes, a future perspective for close collaboration. With this already being a barrier in a generally well-served city, interviewees doubted the feasibility of the COMET concept for other and more rural areas.

In addition, COMET was considered a special construct for a small group of care providers, namely “the sub-group of motivated and committed colleagues who say ‘I’ll get involved beyond my working hours’” (MHP/m/T1/24). As the number of included mental disorders in COMET was limited, MHPs felt a discrepancy to their everyday practice where “[we] of course all treat a much broader field” (MHP/f/T2/18). Faster referrals were due to a privileged access within the trial, not changing but rather “shifting” the underlying central “capacity problems” (MHP/m/T2/13). Even if COMET did refund collaborative actions, such as phone calls to discuss shared patients, this remuneration was considered acceptable under trial conditions but insufficient if paid in usual care. Interviewees did not perceive communication within COMET as significantly improved or different to usual care, although contacts were initiated slightly more often. While outside the COMET study, MHPs were rather reluctant to share information on the intimate issues addressed in psychotherapy, sharing information between psychotherapists and PCPs seemed easier in COMET. One reason was that patients in COMET had all talked to their PCPs about their mental health problems at study inclusion and had been referred by them, thus paving the way for more information exchange between involved care providers. MHPs reported that collaboration was mainly initiated from their side, pitying that there “has never been the impression that there is much interest [on the PCPs’ side] to continuously get information” (MHP/f/T2/17). At the same time, some MHPs did not evoke much need for intensified collaboration, maybe as a result of “being so immersed in my routine” (MHP/m/T2/13). Apart from the trial-associated barriers, further hindrances originated from general, practice and personal circumstances, e.g. vacancies and changes in staff, IT problems, and personal burden.

#### COMET network meetings

The COMET network meetings took place quarterly, most starting with a professional input, followed by interprofessional exchange. They were welcomed for being a room for exchange, personal contacts and interesting input on both care providers’ sides. Frequency and length (each lasting 3 h) were rated as acceptable but still strenuous after a long workday. Although some MHPs judged the input as being mostly addressed at PCPs, the awareness raising among PCPs for mental health issues was acknowledged. Despite the generally positive evaluation, the network meetings were not experienced as events for forging substantial collaboration as there had been, according to both professional groups, too little room for getting to know each other at the beginning of the trial and too little interprofessional exchange during the first of the subsequent meetings. To this adds a perceived fluctuation in participating network members, with a clear tendency of MHPs attending more consistently compared to PCPs. Yet, most but not all interviewees still pledged for continuing the network meetings under the guidance of the University Medical Center.

#### Ideas for improving collaboration

Based on the interviewees’ collaboration experiences in usual mental health care and their mixed evaluation of collaboration within the COMET CSC model, the most prominent idea for improvement of future collaboration was to arrange networks more locally. By assuring sufficient spatial proximity, which would entail more patients being shared by both MHPs and PCPs, networks would have a chance to be more sustainable. Still, participants of both the PCPs and the MHPs group endorsed the idea of carrying forward the COMET network beyond the trial period, with further professional groups. Interprofessional case conferences were repeatedly suggested as a helpful format. With regard to future collaboration within the network (or other collaboration models), the aforementioned aspects resonated: remuneration and time for collaborative activities, and improved reachability, e.g. by digital communication channels.

## Discussion

The present process evaluation of the COMET study shed light on the PCPs’ and MHPs’ perspectives with regard to collaborative care within a newly implemented CSC model, against the background of usual mental health care and collaboration. The usual mental health care situation was described by both the CSC and the TAU group as impaired by insufficient resources, with collaboration mainly taking place, if at all, within informal local networks. Taking this into consideration, the care provided within COMET was appraised as improved due to shorter waiting times, more personal contacts, increased mutual knowledge and slightly improved feedback loops. Yet, collaboration expectations were only partly fulfilled and optimal collaboration, in the interviewees’ eyes, would have consisted of more frequent and more in-depth information exchange, in a denser and more consistent network in close proximity and with more structural changes to allow for collaboration time and remuneration.

The importance of personal contacts, e.g. as enabled by the CSC network meetings, has been identified as a key factor for fostering collaboration, with closer contact in terms of regular team meetings or co-location as even more helpful [17–20]. This reflects the COMET participants’ wish for a more local network instead of the regionally wide-spread COMET network and underlines the specific challenges of collaborating in independent outpatient practices. This way of rather “horizontal integration” substantially builds on the participants’ willingness to collaborate and stands in contrast to the more “vertical integration” in hospitals or overarching health organizations, where e.g. leadership can enhance collaboration [23, 36]. Despite the variety of pathways that led to the creation of informal networks, personal contacts were central to most ways of connection. A key question, subject to differences in health care systems, is to what degree these personal contacts have to be formalized, how close collaborative care needs to be and how evidence-based collaborative care models can be implemented and aligned with health care specifics [37]. In line with previous research [19, 20], COMET PCPs and MHPs welcomed the benefits of participating in a network of committed care providers, sharing the goal of better caring for patients. Especially in the COMET PCPs’ eyes the study succeeded in allowing for shorter referral pathways, congruent to previous studies on collaborative and stepped care that solely focused on depression and on somatoform disorders [21, 31]. While in COMET communication and feedback loops only slightly improved despite the participants’ wish, this represented an important component of collaboration in a systematic qualitative review by Overbeck et al. [20] and should be addressed in future CSC models. Helpful strategies, according to the participants, could include systematic feedback rules, e.g. by setting fix time points in the treatment process for mutual reports, remuneration for exchange activities or the development of acceptable digital communication channels, such as secure email systems or shared electronic records. This has to be considered against the backdrop of the overall mental health care problems with lack of resources, deficits in remuneration of collaboration and high workload. These facts prominently emerged in the COMET study as inhibiting factors of the participants’ commitment and collaboration, concordant with previous trials [19–21].

In addition to these structural barriers that impeded on the implementation of COMET, interprofessional differences rose up repeatedly and as a major barrier to collaborate in such a “horizontal” [36] collaboration setting. While role definitions were partly clear as to which tasks were primarily realized by either PCPs or MHPs, several conflicts arose: e.g. the doubts of MHPs with regard to PCPs’ mental health competencies, the responsibility for caring for mild to moderate mental disorders, the felt lack of esteem on the MHPs’ side and the impression on the PCPs’ side that MHPs partly were reluctant to cooperate. These cultural differences and divergences in interests between the professional groups, also described as “territoriality” [36], were equally cited as counterproductive in studies realized in other countries [20, 38]. In previous research and in COMET [20, 39], both PCPs and MHPs worried about their autonomy and status. At the same time, in COMET and in other studies, MHPs have been identified as the professional group more likely to be subdued when care is advancing towards integrated care, as this is mostly realized within the medical system [40]. To counteract the tendency towards “territoriality”, both provider groups have to be involved on equal level, paving the way towards a more “altruistic” stance to collaboration [36]. Examples of how this might be realized in mental health care are accounting for the competencies of all care provider groups in designing stepped care pathways and in deciding on whether to step up or down treatment or adequately paying the services provided by all professional groups, especially regarding consultation time and collaborative activities. To diminish interprofessional difficulties, and in addition to the identified personal encounters as helpful, further strategies can be applied to increase mutual understanding, develop a shared language and treatment approach. These include e.g. interdisciplinary further training as already researched on in physicians and nurses [41], well-embedded digital exchange channels [42] and interprofessional case conferences [43]. Existing international guidance on how to foster collaborative practice and interprofessional education [44, 45] should be consulted and adapted to the specific setting, such as care networks without co-location.

Taken together, the present process evaluation of the COMET trial reveals the importance of taking into consideration the different care provider roles, of creating room for interprofessional encounter in local networks and of improving general mental health care conditions in order to foster collaborative care in usual care settings for the most common and comorbid mental disorders. Collaborative care, if realized, requires effort but can improve mental health care substantially.

### Strengths and limitations

The results of the present process evaluation add to the current knowledge by examining the implementation of a CSC model closely aligned to usual mental health care without additional health care professionals, such as care managers or study nurses. Moreover, collaboration experiences within the CSC model were explicitly embedded in the usual mental health care experiences of the involved care providers, thereby anchoring the results in the overall health care situation. Study results have to be considered in light of the following strengths and limitations. Adopting a qualitative approach allowed for capturing in depth the experiences of the participants, especially as we conducted interviews at two time points and could thus retrace the changes in collaboration. Collaboration was investigated from both sides of the collaborating professions, i.e. PCPs and MHPs, and the sampling procedure aimed at further increasing heterogeneity within the sample. Since both the COMET trial participation and the interview participation relied on the participants’ willingness to engage in research, a self-selection bias towards health care providers who were interested in collaboration can be assumed. Answers might have been influenced by social desirability and by a self-bias with regard to the evaluation of the care providers’ own work. As to the process of interviewing and analyzing the results, the researchers’ background and study involvement might have further introduced biases. With regard to interviewing, the conducting researcher (SW) transparently communicated the potential role conflict during the interviews and encouraged the interviewees to speak openly. Being a sociologist helped in eliminating professional role overlap with the care providers involved. As to the process of analysis, regular discussions and contrasting of coding and interpretations between SW and KM and within the COMET study team and the affiliated research institutions aimed at increasing awareness of potential biases due to study involvement and professional affiliation. However, one bias may be that both researchers closer aligned to the care providers’ and patients’ side than taking a systems’ approach, e.g. with a focus on cost-effectiveness.

As we explicitly decoupled the process evaluation from the main study results to prevent priming of participants’ experiences, our study results certainly only contribute one important column for the global evaluation of the COMET trial as an exemplary CSC model. While COMET is grounded in the German health care system and the Hamburg region, we believe the identified challenges and facilitating factors are of general relevance to mental health care. Difficulties in collaboration e.g. due to diverging and competing professional identities and health care settings, due to sector barriers, communication problems as well as general deficiencies in health care resources and remuneration structures also arise in health care systems differing from the German one and the care situation in Hamburg. Thus, our results can, at least to a certain extent, be transferred to other countries and regions, especially to care provided without co-location. Although the present study provides insights into collaboration in CSC models, further research is needed as to the sustainability of such models outside of trials, as this has been identified as challenge [46].

## Conclusions

The present process evaluation of the care providers’ perspectives in the COMET trial shed light on important aspects that have to be considered in future research and implementation of collaborative care models. While it is indispensable to take into account the main trial results and the patients’ perspective (to be published) to gain an overall picture, further research and practice recommendations can be deduced from the present results: 1) Future collaboration models should be implemented and examined in reasonable local boundaries, to allow for personal contact, as this has been one major benefit within the COMET trial. Moreover, further professional groups should be involved, as well by capitalizing on the potentials of tele-healthcare and digital communication. 2) Faster patient referral pathways, supported by a digital booking tool, have proven essential for improved patient care and a relief for PCPs in COMET. This should equally be fostered in future CS models. 3) Considering the prominent role of professional delineations and questions of power and competence that might impede on patient care, further research should be conducted on how to overcome these delineations, especially in settings without co-location, in favor of an interplay of competencies rather than opposition.

## Supplementary Information

**Additional file 1.** Interview guides for the COMET process evaluation.

**Additional file 2.** Summary of the main results and exemplary quotes on collaboration within the COMET trial.

## Data Availability

The datasets generated and analyzed during the current study are not publicly available due to the traceability of individual participants, but available from the corresponding author on reasonable request.

## Abbreviations

CBT: Cognitive-behavioral therapy
CC: Collaborative Care
CME: Continuing medical education (required for care providers)
COMET: Collaborative and Stepped Care in Mental Health by Overcoming Treatment Sector Barriers (trial name)
CSC: Collaborative and Stepped Care
MHP: Mental health professional(s)
PCP: Primary care physician(s)
PD: Psychodynamic psychotherapy
RCT: Randomized-controlled trial
TAU: Treatment as usual
