# Supplementary material for: How do care providers evaluate collaboration? - qualitative process evaluation of a cluster-randomized controlled trial of collaborative and stepped care for patients with mental disorders
Source: BMC Psychiatry. 2021 Jun 8;21:296. doi: 10.1186/s12888-021-03274-3 (PMC8184353; doi:10.1186/s12888-021-03274-3)
Supplement: Supplementary file 1 — Additional file 1. Interview guides for the COMET process evaluation. [file 12888_2021_3274_MOESM1_ESM.docx]

## How do care providers evaluate collaboration? - Qualitative process evaluation of a cluster-randomized controlled trial of collaborative and stepped care for patients with mental disorders

Kerstin Maehder / Silke Werner, Angelika Weigel, Bernd Löwe, Daniela Heddaeus, Martin Härter / Olaf von dem Knesebeck

**Additional file 1. Interview guides for the COMET process evaluation**

**Abbreviations**

COMET Collaborative and Stepped Care in Mental Health by Overcoming Treatment Sector Barriers (trial name)

CSC Collaborative and Stepped Care (trial care model)

TAU Treatment as usual (trial control group)

**Interview guide for primary care practitioners on collaboration at T1:**

**COMET CSC group & TAU group**

1. How do you evaluate the current health care situation for patients with mental disorders?
2. How would you describe the role of your PCP practice in health care for patients with mental disorders?
   1. How do you perceive yourself as general practitioner? What are your strengths and weaknesses? What kind of support do you wish for?
   2. Coming back to the difficulties in mental health care described in 1.: How do you cope with these?
3. Why did you chose to participate in the COMET study?
4. Do you have personal contacts to mental health care providers (psychotherapists/ psychiatrists/ inpatient clinics)?
   1. How would you describe your communication, collaboration and networking with these?
   2. Would you describe these contacts as a network?
   3. If so, what are the characteristics of this network?
5. What do you expect from the COMET study, especially regarding collaboration?

**Interview guide for primary care practitioners on collaboration at T2:**

**COMET CSC group & TAU group**

1. Has anything changed during the last year as regards your evaluation of the current health care situation for patients with mental disorders? *(presenting answers from T1)*
2. Has anything changed during the last year as regards you collaboration with psychotherapists, psychiatrists and inpatient clinics? (outside and within the COMET study)
3. ***Questions in the following only for intervention group:***

How would you evaluate collaboration within the COMET study?

- 1. Were you satisfied with the care provided by the COMET mental health professionals?
  2. How would you evaluate the online appointment platform?
  3. What went well within the COMET network? What could be improved?
  4. How did communication within the COMET network work? (communication channels, information exchange)
  5. Did you participate in the COMET network meetings? Why/why not? How would you evaluate the course and content of the network meetings?
  6. What would you wish for regarding collaboration within the COMET network in the future? (frequency and content of network meetings, further network partners, needs, sustainability, communication)

**Interview guide for mental health professionals on collaboration at T1:**

**COMET CSC group & usual care outside COMET**

1. How do you evaluate the current health care situation for patients with mental disorders?
2. How would you describe the role of your mental health care practice in health care for patients with mental disorders?
3. How do you perceive the intersection with primary care?
4. How do you perceive yourself as mental health professional? What are your strengths and weaknesses? What kind of support do you wish for?
5. Coming back to the difficulties in mental health care described in 1.: How do you cope with these?
6. Why did you chose to participate in the COMET study? (only intervention group)
7. Do you have personal contacts to other care providers?
   - 1. How would you describe your communication, collaboration and networking with these?
     2. Would you describe these contacts as a network?
     3. If so, what are the characteristics of this network?
8. What do you expect from the COMET study, especially regarding collaboration? (*only intervention group*) resp. What do you think about the aims of the COMET study? (*only control group*)

**Interview guide for mental health professionals on collaboration at T2:**

**COMET CSC group**

1. Has anything changed during the last year as regards your evaluation of the current health care situation for patients with mental disorders? *(presenting answers from T1)*
2. Has anything changed during the last year as regards you collaboration with other care providers? (outside and within the COMET study)
3. How would you evaluate collaboration within the COMET study?
   - 1. Were you satisfied with the care provided by other COMET care providers?
     2. How would you evaluate the online appointment platform?
     3. What went well within the COMET network? What could be improved?
     4. How did communication within the COMET network work? (communication channels, information exchange)
     5. Did you participate in the COMET network meetings? Why/why not? How would you evaluate the course and content of the network meetings?
     6. What kind of information would you like to have from primary care physicians? What kind of information could they need from you?
     7. What would you wish for regarding collaboration within the COMET network in the future? (frequency and content of network meetings, further network partners, needs, sustainability, communication)
