# Supplementary material for: How do care providers evaluate collaboration? - qualitative process evaluation of a cluster-randomized controlled trial of collaborative and stepped care for patients with mental disorders
Source: BMC Psychiatry. 2021 Jun 8;21:296. doi: 10.1186/s12888-021-03274-3 (PMC8184353; doi:10.1186/s12888-021-03274-3)
Supplement: Supplementary file 2 — Additional file 2. Summary of the main results and exemplary quotes on collaboration within the COMET trial. [file 12888_2021_3274_MOESM2_ESM.docx]

## How do care providers evaluate collaboration? - Qualitative process evaluation of a cluster-randomized controlled trial of collaborative and stepped care for patients with mental disorders

Kerstin Maehder / Silke Werner, Angelika Weigel, Bernd Löwe, Daniela Heddaeus, Martin Härter / Olaf von dem Knesebeck

**Additional file 2. Summary of the main results and exemplary quotes on collaboration within the COMET trial**

Table 1 Summary of the main results and exemplary quotes on collaboration within the COMET trial, against the background of usual mental health care

| Subcategories | Themes | Details | Exemplary quotes |
| --- | --- | --- | --- |
| 1) Usual mental health care | | | |
| Evaluation of usual mental health care | Insufficient mental health care offer | Unanimously judged as insufficient by PCPs and MHPs, entailing risks for e.g. chronification. | - „[The mental health care situation is] dramatic“ (MHP/f/T1/17) - “It still takes a lot of time until you get someone treated somewhere [in mental health care], you already rejoice if you get an appointment in one or two month for a first consultation” (PCP/m/T1/5) - “[The main problem is] that it takes far too long until patients are treated adequately. And that it [the disorder] becomes chronic and that long periods of inability to work develop.” (PCP/m/T1/6) |
| COMET trial motivation & expectations | Improving collaboration as main motivation | Increased collaboration as main trial motivation for both PCPs and MHPs. | - „And I found the idea of networking attractive […], we really do this way too little. I do not know a single colleague who has intensive contact to primary care physicians”. (MHP/f/T1/5) |
|  | Structured care pathways | PCPs wishing for more guidance in form of structured care pathways for mental disorders. | - “That you get something like a guideline that you can walk along, without it being too narrow. A practicable model that I can rely on. (PCP/f/T1/1) |
|  | Minor sources of motivation | Less frequently mentioned sources of motivation, e.g. financial incentives, CME credits, interest in research. | - “To be honest, all that thing about money [the incentives], that’s somewhat nice but that’s not why one would take part in a study.” (MHP/f/T2/6) |
| 2) Collaboration in usual mental health care | | | |
| Care providers‘ roles | PHPs’ role | Examples of PCPs’ assumed role in mental health care: first point of contact, coordinating care, trustful long-term relationships, medication, sick certification. | - “I consider my job as one of being an attendant and as being a pilot [in the health care system]” (PCP/m/T1/4) - “Many [patients] who come back and where you really do primary care work. In that we follow them through their whole lives, often know their families, know their crises, that partly have emerged from illnesses, like cancer, know their social environment.” (PCP/m/T1/8) |
|  | Barriers for PHPs in mental health care | Lack of time, high number of patients, lack of mental health competence | - „On the other hand, I don’t really have the patience to look for the causes [of mental disorders], by talking or something like that, because this simply isn’t possible with having 60 to 100 patients here on a morning.” (PCP/m/T1/6) |
|  | MHPs’ role | Examples of MHPs’ assumed role in mental health care: differential diagnostics, psychoeducation, treatment | - “To first assess ‘Do they [the patients] need psychotherapy or something else? Is outpatient treatment possible or do they need to get inpatient treatment first?’” (MHP/m/T1/12) |
|  | Barriers for MHPs in mental health care | Need for resp. lack of mutual fit between therapist and patient, felt lack of esteem from physicians | - “We work with our relationship, it is impossible that I get forced to take just any patient” (MHP/f/17) - “I still have the feeling that there is not much appreciation. And that most physicians don’t even know what we do and how much it matters to patients that there is someone listening” (MHP/f/T1/19) |
| Evaluation of collaboration in usual mental health care | Insufficient collaboration in usual mental health care | Overall collaboration in usual mental health care unanimously judged as insufficient; collaboration mainly taking place in small informal networks | - “Contacts to primary care physicians are scarce, really, really scarce” (MHP/f/T1/3) - “It all depends on your own network and whether you’re willing to pick up the telephone […]” (PCP/f/T1/10) |
|  | Barriers to collaboration | Lack of time, resources, financial remuneration, personal contacts and willingness, intimacy of topics addressed in psychotherapy | - “The problem is that it gets too much in everyday practice and that too much has to be decided on quickly and I would like to have more time for referral […] but phoning, faxing, writing, that all costs much time. Which would be taken from the patients.” (PCP/f/T1/3) - “In many cases, it’s the patient who asks for limited transfer of information to the PCP” (MHP/m/T2/1) |
|  | Benefits of collaboration | shorter referrals, coordinated care, increased knowledge | - “With all colleagues from that very psychiatric practice, there’s close exchange about patients and that really improves care” (MHP/f/T1/5) - “And then there is a psychotherapist with whom I’m in good contact and when I really do have [a patient] who needs a crisis intervention, then I call her, describe the case and she’s actually always willing to take someone on short notice.” (PCP/f/T1/1) |
| 3) Collaboration within COMET trial | | | |
| Evaluation of collaboration within COMET | Benefits of collaboration within COMET | Increased collaboration and mutual understanding, more personal contacts, easier & faster referrals from PCPs to MHPs, COMET-patients partly perceived as more reliable | - “What has improved in any case is that I systematically think of sending at least short notes [to the PCPs]. Sure, there still is room for improvement […] but communication has improved in terms of more exchange.” (MHP/m/T2/2) - “There haven’t only been quicker referrals and the handing over from our side, but the feedback [improved as well]. (PCP/m/T2/4) |
|  | Main barriers for collaboration within COMET | Lack of time, communication and mutual reachability still insufficient, unfavorable regional distribution of COMET network, COMET-patients partly perceived as less motivated, concept not sustainable | - “It’s different, the direct surroundings, where ways are short and you know the providers. That’s different [from COMET] when you have to tell your patient ‘You just have to drive to the other end of the city’”. (PCP/m/T2/5) - “We [a colleague and me] both made the experience that the patients [in COMET] were clearly less motivated and had less appreciation for such a [therapy] offer, maybe because they got it so easily.” (MHP/f/T2/5) |
| COMET network meetings | Benefits of COMET network meetings | Increased personal contact & mutual understanding, helpful input, raising PCPs’ awareness for mental health | - “I didn’t think about what a day looks like for a PCP, I was just irritated when I again tried to reach one for hours. (…) Within the group work [at the network meetings] I understood some of the difficulties that PCPs have to struggle with in their everyday practice” (MHP/m/T2/14) |
|  | Barriers for COMET network meetings | Additional work burden, input mostly relevant for PCPs, lack of consistent participation & network building | - “It [the network meeting] hasn’t brought about closer contacts. There are always different people and you probably work at the very opposite end of the city or there are again mainly psychotherapists and little primary care physicians.” (MHP/f/T2/3) - “Sometimes there was quite a disappointing feeling because for two hours in the evening I listened to something on anxiety disorders, which, as psychotherapist, you usually already learned at university. ” ((MHP/f/T2/5) |
| Ideas for improving collaboration | More localized and extended networks | Creating more localized networks, including further professional groups, regular case conferences 🡪 promoting sustainability | - “I think if you were to repeat such a study, you would have to take a more regional approach, that you really work to create small networks locally.” (MHP/f/T2/6) |
|  | Improving overall health care conditions | More time, resources and financial remuneration for collaboration | - “In the end, [COMET] is just some kind of shifting. In that a certain group of patients now gets categorized as being urgent and gets favored over others. But this doesn’t change anything in terms of basic care structures.” (MHP/m/T2/1) |

COMET = name of the trial

MHP = Mental health professionals

PCP = Primary care physicians

Quotes were edited for legibility and explanations have been added in square brackets where necessary. Quotes are identified by professional group (PCP vs. MHP), gender (female (f) vs. male (m)), T1 or T2 interview and number of participant.
